# Genetic risk scores may compound rather than solve the issue of prostate cancer overdiagnosis

**DOI:** 10.1038/s41416-022-02080-2

**Published:** 2022-11-28

**Authors:** Rachel H. Horton, Malcolm G. Dunlop, Richard S. Houlston, Anneke Lucassen, Margaret McCartney, Alan McNeill, Amit Sud

**Affiliations:** 1 Clinical Ethics and Law, Faculty of Medicine, University of Southampton, Southampton, UK; 2 Wessex Clinical Genetics Service, Princess Anne Hospital, Southampton, UK; 3 Wellcome Centre for Human Genetics, University of Oxford, Oxford, UK; 4 Centre for Personalised Medicine, University of Oxford, Oxford, UK; 5 Colon Cancer Genetics Group, Medical Research Council Human Genetics Unit, Institute of Genetics and Molecular Medicine, University of Edinburgh, Edinburgh, UK; 6 Division of Genetics and Epidemiology, The Institute of Cancer Research, London, UK; 7 Population and Behavioural Science Division, School of Medicine, University of St Andrews, St Andrews, UK; 8 Edinburgh Urological Cancer Group, University of Edinburgh, Western General Hospital, Edinburgh, UK; 9 School of Engineering and Physical Sciences, Heriot Watt University, Edinburgh, UK; 10 Haemato-oncology Unit, The Royal Marsden Hospital NHS Foundation Trust, Sutton, UK

**Keywords:** Predictive markers, Risk factors, Cancer screening, Urological manifestations, Population genetics

We read with interest the recent article by Green et al., where the authors assert that use of genetic risk scores could inform decisions in primary care as to whether to investigate men with lower urinary tract symptoms for prostate cancer [1]. However, we contend that such genetic risk scores are only weakly predictive and using them prematurely in clinic may create more problems than it solves.

The authors used an integrated risk model including genetic risk score and age for men whose GP records indicated lower urinary tract symptoms (LUTS), looking at the ability of the model to predict which men would be diagnosed with prostate cancer in the two years after their symptoms were first recorded. With the model set to identify men at 3.7% or higher chance of being diagnosed with prostate cancer within two years (the point at which, from a statistical perspective, the model achieved the optimal balance between sensitivity and specificity), this achieved a detection rate of 71%. That is, within a population of men with lower urinary tract symptoms, 71% of those who would be diagnosed with prostate cancer by ‘traditional’ referrals within two years would have a ‘positive score’ on the integrated model, whilst 29% would be missed. However, 31% of the men who would not go on to be diagnosed with prostate cancer over the next two years would also have a positive score and therefore be referred for further investigations based on LUTS and an elevated score.

We argue that the problem of false positives here deserves attention. As the authors note, ‘*evidence for an association between lower urinary tract symptoms and the risk of prostate cancer is equivocal*’, *i.e*. the pre-test probability that a man presenting with such symptoms has prostate cancer is not high. Indeed, NICE recommends that men with lower urinary tract symptoms should be offered ‘*information, advice and time to decide*’ if they wish to have PSA testing, if their symptoms are suggestive of bladder outlet obstruction secondary to benign prostate enlargement; their prostate feels abnormal on digital rectal examination; or they are concerned about prostate cancer [2]. Yet PSA is more predictive of prostate cancer metastasis or death than a genetic risk score [3], and if the benefits of PSA in the context of lower urinary tract symptoms are evidently debatable, how is a weaker test going to help? Rather than improving triage of men with these symptoms, our concern is that while from a technical perspective genetic risk scores may very slightly improve prostate cancer prediction, the price of a marginal increase in diagnoses of (sometimes indolent) prostate cancer will be that more people will be swept along a pathway of ultimately unnecessary invasive investigations, while a small number will be falsely reassured.

In general, genetic risk scores for prostate cancer are designed to detect cancer incidence, rather than aggressive cancer, reflecting the predominant focus of the genome-wide association studies from which these scores are derived. The high incidence of indolent prostate cancer represents a hurdle that genetic risk scores have yet to evade [3], and the authors recognise this, writing that ‘*Identifying aggressive prostate cancer is a key focus of prostate cancer diagnosis research efforts; this could not be assessed in the present study due to a lack of cancer stage data*’. Another major issue that the authors rightly point out is that black men are twice as likely to present with advanced-stage prostate cancer [4, 5], but this analysis only included white European men because of the lack of diversity in the UK Biobank on which this study was undertaken. This is an issue across the field, rather than specific to this study, but it means that genetic risk scores stand to be less useful to black men, who are already disproportionally affected by prostate cancer.

The poor performance of genetic risk scores at detecting life-limiting prostate cancer, and the inequity they stand to worsen if they work, are noted in the article, but these represent fundamental barriers which need addressing before such scores are fit for clinical use. We recognise that prostate cancer investigation is fraught with challenges – PSA tests are unreliable, and distinguishing men who might die from prostate cancer from men who will die with it, is often difficult. Of course we need to explore options that might improve this situation, but guiding decisions around PSA testing by using an even weaker test is unlikely to represent a major advance.
